# Perceived Risks of Infection, Hospitalization, and Death From COVID-19 at the Equator: Ecuador and Kenya

**DOI:** 10.1017/dmp.2021.268

**Published:** 2021-08-16

**Authors:** Tullaya Boonsaeng, Carlos E Carpio, Patricia Guerrero, Oscar Sarasty, Ivan Borja, Darren Hudson, Anthony Macharia, Mumina Shibia

**Affiliations:** 1 Department of Agricultural and Applied Economics, Texas Tech University, Lubbock, Texas, USA; 2 Departament of Biochemistry, Faculty of Science, University of Zaragoza, Zaragoza, Spain; 3 Department of Business Administration, Ana G Mendez University, San Juan, Puerto Rico; 4 Kenya Forestry Research Institute, Nairobi, Kenya; 5 Kenya Agricultural and Livestock Research Organization, Nairobi, Kenya

**Keywords:** covid-19, Ecuador, Kenya, cross-sectional studies

## Abstract

**Objectives:**

This study’s goal was to determine the perceived risks of infection as well as the perceived risks of hospitalization and death from COVID-19 in Ecuador and Kenya. It also assessed the factors associated with the risk-related perceptions.

**Methods:**

Cross-sectional studies with samples from the adult populations in both countries were conducted to assess the perceived risks of contracting COVID-19. Data were collected online using the Qualtrics platform (Qualtrics, Provo, Utah, United States) from samples of 1050 heads of households, aged 18 years or older, in each country. A total of 3 statistical analyses were conducted: summary statistics, correlation, and linear regression.

**Results:**

The average perceived risks of COVID-19 infection, hospitalization, and death in the Kenyan sample were 27.1%, 43.2%, and 17.2%, respectively, and the values for the Ecuadorian sample were 34%, 32.8%, and 23.3%, respectively. The Pearson’s correlation coefficients between the risk measures in each country were less than 0.38. Risk measures were associated with several sociodemographic variables (e.g., income, gender, location), but not with age.

**Conclusions:**

The perceived risks of COVID-19 infection, hospitalization, and death in Kenya and Ecuador were significantly higher relative to the statistics reported; however, no strong association existed between perceived risk and age, which is a key factor in adverse health outcomes, including death, among COVID-19 infected individuals.

## Introduction

On March 11, 2020, the coronavirus disease 2019 (COVID-19) was declared a pandemic by the World Health Organization and has since affected people and economies worldwide.^1^ Some experts classify COVID-19 as a syndemic, which refers to a disease that interacts with others, putting another burden on the population.^2^ By June 19, 2021, The New York Times Coronavirus vaccine tracker listed 8 vaccines approved for full use, 94 vaccines in human trials, and at least 77 vaccines in animals.^3^ Simultaneously, several countries are suffering a second or third COVID-19 wave, and in controlling the disease, have to continue relying on individual health measures: social distancing, masks, and washing hands often. Individuals’ adherence to these actions depends on their perceptions of infection, hospitalization, and death risks if they contract COVID-19.^4,5^

This study was carried out to determine the perceived risks of infection, as well as the perceived risks of hospitalization, and death related to COVID-19 in Ecuador and Kenya, and to assess the factors associated with these risk perceptions. Ecuador and Kenya are middle-income countries on the Equator but in different continents with large differences in the impacts of COVID-19 and different population sizes. By June 19, 2021, Ecuador had reported over 440000 confirmed cases and over 21000 deaths from a population of 17.6 million; Kenya had suffered over 177000 confirmed cases and over 3400 deaths from a population of 53.8 million.^6^ Estimated case-fatality rates by June 19, 2021 were 4.8% for Ecuador (8^th^ largest in the world), and 1.9% in Kenya (about 78^th^ in the world). It is also reported that Ecuador experienced 1 of the worst outbreaks during the first months of the pandemic (March-April 2020).^7^

A literature review on Google Scholar identified many studies related to COVID-19 in Ecuador and Kenya (several hundred in each country). In Ecuador, a large share of the literature is devoted to clinical/biological studies.^8–10^ In Kenya, most research efforts appear to focus on the pandemic’s effects on multiple health and economic outcomes.^11,12^ Thus, a limited number of studies are related to the behavioral aspects of the pandemic, particularly on COVID-19 related perceived risks.^13,14^ Moreover, previous assessments of COVID-19 risks have focused only on the perceived risk of infection.^15,16^ However, behavioral responses to preventing the disease may also be related to risk perceptions of adverse health outcomes and even death, if infected.

## Methods

### Study design and survey instrument

Cross-sectional studies were conducted in both countries to assess the perceived risks of COVID-19. Data were collected online using Qualtrics (Qualtrics, Provo, Utah, USA) from samples of their adult populations.^17^ 1050 respondents were selected randomly from market research panels in each country. The sample size was determined to provide mean perceived risk estimates with an approximate relative error of 5% and 95% confidence.^18^ The sample size was also deemed appropriate to conduct regression analyses.^19^

The surveys were designed to have samples that matched the income and household size distribution in each country’s population.^20–24^ Data were collected from April 2 to April 7, 2020, in Ecuador, and from April 7 to April 15, 2020, in Kenya. An initial screening question asked respondents whether they were 18 years older and household heads. Only those who answered ‘yes’ to this question could continue with the survey. Age and household head status were double-checked using questions included in the demographic characteristics section.

The structures of the surveys were the same for both countries. However, questions were tailored to reflect each country’s unique conditions (e.g., names of territorial divisions). The study authors, who include collaborators from the United States, Kenya, and Ecuador, helped design the surveys (English in Kenya and Spanish in Ecuador). All of the authors have extensive experience in survey development. Out of the authors, 2 who are bilingual (Spanish/English), developed the initial versions of the surveys. The collaborators in each country subsequently reviewed the surveys. A pilot test with 100 individuals in each country was used to assess survey completion time, identify problematic questions, and check whether the sample size was appropriate for statistical analyses.

### Measures

The study’s 3 outcomes were perceived risks of infection, hospitalization, and death if infected with COVID-19. The researchers asked 3 questions: (1) What do you consider is your probability of contracting COVID-19?, (2) What do you consider is your probability of being hospitalized after contracting COVID-19?, and (3) What do you consider is the mortality rate in cases of contracting COVID-19? (i.e., the percentage of people who die after contracting the disease). The responses were assessed using a 0 to 100% scale.^15,16^

The explanatory variables were the sociodemographic characteristics of age, monthly income, employment status, gender, education, health insurance, location (urban and rural), and the region of residence. Kenya’s health coverage is very low, and only 20% of its population has health insurance.^25^ Ecuador has a higher level of health insurance coverage, but it is still relatively low (approximately 40%).^26^ Participants were classified into 4 areas of residence in Ecuador (Pichincha Province, Guayas Province, the Sierra and Amazon regions, and Costa and Galapagos regions); these include the 2 most populated provinces, Guayas and Pichincha (where the capital, Quito is located), and the traditional geographical regions. In Kenya, participants were classified according to 3 areas of residence: Nairobi, Rift Valley, and Other. The Nairobi region includes the capital, Nairobi, and the Rift Valley is the largest and most populated area in the country.

### Statistical analysis

A total of 3 statistical analyses were conducted. Basic summary statistics were calculated first (means, medians, and standard deviations). Correlation analysis between the perceived probability measures was then carried out to study their strength of association. Finally, linear regression models were estimated to assess the relation between the explanatory variables (10 in Kenya, and 9 in Ecuador) and the 3 perceived risk probabilities.^19^ Heteroscedasticity consistent standard errors were used. All analyses were performed using the statistical software STATA (StataCorp., College Station, Texas, USA). Separate analyses were conducted for each country.

## Results

### Summary statistics

Due to incomplete data from the original 1050 households in both countries, 972 and 963 observations remained for Ecuador and Kenya, respectively. The Kenyan respondents’ mean monthly household income was KES (Kenyan Shillings) 17539.98 (US$163.13 at 107.52 KES = 1.00 USD). Most respondents were male (63%), college-educated (87%), had health insurance (72%), and employment (73%), and lived in urban areas (76%); their mean age was 30 years. The majority (39%) lived in the Nairobi region (Table 1).

With respect to the Ecuadorian respondents, their mean monthly household income was $827.29. Like the Kenyans, most were male (61%), had some college education or above (72%), health insurance (58%), and employment (74%), and lived in urban areas (86%). Most resided in Pichincha Province, where the capital of Quito is located. Their mean age was 33 years.

### Perceived risks of covid-19 and associated variables

The mean perceived risks of COVID-19 infection, hospitalization, and death in the Kenyan sample were 27.1%, 43.2%, and 17.2%, respectively, and the median values for the 3 measures were 20%, 35%, and 12%. The Pearson’s correlation coefficient between the risk of infection and hospitalization was 0.38, between the risk of infection and death was 0.32, and between the risk of hospitalization and death was 0.25 *P* <0.05 for all correlations). The perceived risk of COVID-19 infection was associated with having health insurance, the perceived risk of hospitalization was associated with having health insurance, while the perceived risk of death was associated with income, gender, location, and region of residence.

The estimated averages (medians) for COVID-19-related perceived risks of infection, hospitalization, and death in Ecuador were 34% (30%), 32.8% (27%), and 23.3% (20%), respectively. The estimated correlation coefficients (*P* < 0.05) between the perceived risk outcomes were 0.33 between perceived risk of infection and hospitalization, 0.08 between perceived risk of infection and death, and 0.11 between perceived risk of hospitalization and death. As shown in Table 2, the perceived risk of contracting COVID-19 was associated with income, gender, location, and region of residence. The perceived risk of hospitalization was associated with gender, education, health insurance availability, and region of residence. Finally, the perceived risk of death was associated with income and gender.

**Table 1.** Characteristics of survey respondents

Characteristic	Mean (Standard Deviation)	
	Ecuador	Kenya
Sample Size	972	963
	Mean (Standard deviation)	
Age (Years)	33.21 (11.43)	30.05 (7.94)
Monthly Income ($)	827.39 (711.36)	163.13 (121.24)
	Category Percentage	
Gender (Female = 1, Male = 0)		
Female	39%	37%
Male	61%	63%
College Education (Yes = 1, No = 0)		
Not college graduated	28%	13%
College graduated	72%	87%
Health Insurance (Yes = 1, No = 0)		
No	42%	28%
Yes	58%	72%
Employment Status (Employed = 1, Other = 0)		
No	26%	27%
Yes	74%	73%
Location of Residence (Rural = 1, Urban = 0)		
Rural	14%	24%
Urban	86%	76%
Region of Residence		
Pichincha^a^	42%	
Guayas^a^	18%	
Sierra and Amazon^a^	22%	
Costa and Galapagos Region^a^	17%	
Nairobi^b^		39%
Rift Valley^b^		18%
Other regions^b^		43%
Perceived risk of infection (%)	33.96%	27.10%
Perceived risk of hospitalization (%)	32.77%	43.22%
Perceived risk of death (%)	23.25%	17.21%

Note: ^a^ indicates characteristic collected only in Ecuador and ^b^ indicates characteristic collected only in Kenya

**Table 2.** Results of linear regression analyses showing factors associated with perceived risks of contracting covid-19 in Ecuador and Kenya

	Contracting Probability		Hospitalized Probability		Death Probability	
	Parameter (95% CI)	*P*-Value	Parameter (95% CI)	*P*-Value	Parameter (95%)	*P*-Value
			Panel A. Ecuador			
Age	0.027 (−0.121, 0.175)	0.721	0.022 (−0.149, 0.194)	0.798	0.082 (−0.020, 0.184)	0.114
Monthly Income ($100)	0.272** (0.032,0. 511)	0.026	−0.134 (−0.413, 0.145)	0.346	−0.416** (−0.574, −0.257)	< 0.001
Gender (Female = 1, Male = 0)	−4.591** (−7.711, −1.471)	0.004	−4.498** (−7.991, −1.005)	0.012	2.907** (0.693, 5.121)	0.010
College Education (Yes = 1, No = 0)	3.142* (−0.489, 6.773)	0.090	3.697* (−0.683, 8.076)	0.098	−0.667 (−3.316, 1.982)	0.621
Health Insurance (Yes = 1, No = 0)	1.129 (−2.132, 4.390)	0.497	4.275** (0.459, 8.091)	0.028	−1.050 (−3.477, 1.378)	0.396
Employment Status (Employed = 1, Other = 0)	−0.781 (−4.561, 2.998)	0.685	−1.342 (−5.939, 3.255)	0.567	−2.140 (−4.905, 0.625)	0.129
Location of Residence (Rural = 1, Urban = 0)	−3.735* (−8.064, 0.595)	0.091	−3.331 (−8.303, 1.641)	0.189	3.110* (−0.040, 6.259)	0.053
Region of Residence (Ref Costa and Galapagos)						
Pichincha	−2.884 (−7.121, 1.353)	0.182	−3.169 (−8.118, 1.781)	0.209	−2.844* (−5.931, 0.244)	0.071
Guayas	3.536 (−1.695, 8.768)	0.185	−5.276* (−10.880, 0.327)	0.065	0.574 (−3.062, 4.209)	0.757
Sierra and Amazon	−4.046* (−8.591, 0.498)	0.081	0.083 (−5.494, 5.659)	0.977	−0.838 (−4.268, 2.593)	0.632
R^2^	0.039		0.023		0.064	
F (*P*-value)	3.59 (< 0.001)		2.16 (0.018)		8.00 (< 0.001)	
			Panel B. Kenya			
Age	0.162 (−0.035, 0.360)	0.107	0.003 (−0.270, 0.276)	0.983	−0.031 (−0.144, 0.081)	0.582
Monthly Income ($100)	0.828 (−0.641, 2.300)	0.269	0.417 (−1.723, 2.557)	0.702	−0.833** (−1.647, −0.019)	0.045
Gender (Female = 1, Male = 0)	−1.600 (−4.838, 1.638)	0.333	−2.634 (−7.391, 2.122)	0.277	4.708** (2.816, 6.600)	< 0.001
College Education (Yes = 1, No = 0)	−0.781 (−5.213, 3.651)	0.730	0.062 (−6.829, 6.953)	0.986	−1.828 (−4.473, 0.818)	0.175
Health Insurance (Yes=1, No=0)	4.659** (1.166, 8.153)	0.009	4.594* (−0.819, 10.008)	0.096	1.581 (−0.471, 3.634)	0.131
Employment Status (Employed = 1, Other = 0)	−2.612 (−6.368, 1.144)	0.173	−4.259 (−9.691, 1.174)	0.124	−0.301 (−2.482,1.880)	0.786
Location of Residence (Rural = 1, Urban = 0)	−2.236 (−6.329, 1.856)	0.284	−0.857 (−6.618, 4.905)	0.770	−2.313* (−4.685, 0.060)	0.056
Region of Residence (Ref Other)						
Nairobi	−3.689** (−7.363, −0.015)	0.049	−0.716 (−6.151, 4.719)	0.796	−2.768** (−4.916, −0.620)	0.012
Rift Valley	1.934 (−2.590, 6.457)	0.402	2.626 (−3.753, 9.004)	0.419	−0.674 (−3.327, 1.978)	0.618
R^2^	0.023		0.008		0.039	
F (*P*-value)	2.21 (0.020)		0.83 (0.586)		4.51 (< 0.001)	

Note: **P < 0.05; *P < 0.10

## Discussion

The perceived risks of illness have long been associated with health behavior; however, much of the literature on risk perceptions refers only to the probability of contracting a disease.^5^ This study evaluated perceptions of 3 types of risks: infection, hospitalization, and death. A large proportion of individuals infected with COVID-19 are either asymptomatic (approximately 40%) or have mild symptoms; thus, the probability of infection may provide only limited information about risk perceptions that affect health behaviors.^27^ The degree of association between these 3 risk measures was relatively weak, therefore, they cannot be used as proxies.

Some authors have argued that a more precise measure of risk of illness involves the probability of infection and a measure of the disease’s expected severity.^28^ This new measure involves multiplying the perceived risk of infection by the probability of hospitalization or death (both provide measures of the expected severity of illness). Such a measure can be interpreted as the perceived joint probability of infection and the adverse health outcomes (hospitalization or death). This interpretation is based on the formula for conditional probability: *P*(*B*∣*A*) = *P*(*A*∩*B*)/*P*(A), in which A refers to infection, and B refers to the adverse health outcome (hospitalization or death), ‘∣’ denotes conditional probability, and ‘Ո’ denotes joint probability. Thus, the joint probability of infection and an adverse health outcome is *P*(*A*∩*B*) = *P*(*B*∣*A*)*P(A).^29^ Note that the question about the risk of infection corresponds to the unconditional probability *P*(A), while the questions about the risk of hospitalization and death correspond to conditional probabilities (*P*(*B*∣*A*)). More work would validate these risk perception measures and relate them to more formal conceptual constructs, and individuals’ expectations of the costs of a disease. Consistent with previous COVID-19 studies, each risk perception measure is discussed separately below.

The estimated median values of the risk of infection reported in Ecuador (30%) and Kenya (20%) were similar to those in the United States (32%) but were significantly higher than those in Indonesia (10%): countries where comparable studies had been conducted at approximately the same time using comparable risk elicitation methods. These values are significantly higher than the official rates of infection. The cumulative risks of infection reported by December 12, 2020, were lower than the perceived risks. Ecuador reported 200000 confirmed cases in a population of 17 million, and Kenya reported 19000 cases in a population of 51 million. Similarly, median perceived risks of death in Ecuador (20%) and Kenya (12%) were significatively higher than the reported fatality ratios of 6.9% and 1.7%, respectively (also reported by December 12, 2020). However, it is important to note that official statistics may differ from actual statistics because of limited testing capacity, for example. Also, in both countries, the number of cases estimated using several epidemiological models is significantly higher than the number of confirmed cases.^30^

In short, there is a large difference between the risks of infection and death that governments and researchers have estimated in both countries, and the greater risks individuals in the population perceived. These large perceived risks of infection and adverse health outcomes did not translate into total compliance with health prevention measures. For example, mask use reported by the Institute for Health Metrics and Evaluation (IHME) was estimated at approximately 70% in Ecuador, and 50% in Kenya during the time of data collection (both values are closer to the maximum values reported throughout the pandemic).^31^

In both countries, risk perceptions tended to be homogenous across age groups, as respondents’ ages were not associated with any of the perceived risks. The lack of association between age and hospitalization and death risks could be problematic as, older individuals were more susceptible to the disease at the beginning of the pandemic; however, it is important to note that perceptions of the risks were high overall and the sample tended to be younger, which may have hindered the ability to detect a relation. This finding may also reflect the lack of communication about these risks to the population. While the risk of infection can be reduced by individual behavioral changes, the probability of being hospitalized or dying after contracting the disease depends less upon individual behavior. This finding contrasts with studies from Indonesia and the United States that reported higher perceived risks of infection among individuals in their 20s to 40s relative to older individuals.^15,16^

A consistently negative and significant association between income and the risk of death was found in both countries. This may be related to perceptions of the quality of services available to poorer individuals relative to wealthier individuals. However, the estimated effects were very small. In Ecuador, a $100 increase in income was associated with only a 0.42% reduction in the perceived risk of death, while in Kenya, a $100 increase in income was associated with a 0.83% reduction in the perceived risk of dying. Larger, significant, and consistent associations were found between gender and risk perceptions in both countries. Relative to men, women’s perceived risk of dying was 2.9% and 4.7% higher in Kenya and Ecuador, respectively. Similarly, women’s perceived risk of contracting the disease was 1.6% and 4.6% lower than men in Kenya and Ecuador, respectively (significant only in Ecuador). Previous studies that have evaluated COVID-19-related risk perceptions found no differences between genders or did not consider this variable relevant.^16^ At the beginning of the pandemic, little was known about differences in the associations between gender, infection, and mortality.^15^ A recent study with data from 17 countries reported a higher infection rate among women (+15%), but higher fatality rates among men (+50%).^32^ It is possible that women’s final exposure was greater than expected initially, given their role as primary homemakers in charge of procuring food for the household and caring for the sick. Some research has also shown that women in developing countries were less likely to have jobs that could be conducted remotely.^33^

Large differences in risk perceptions between locations and regions were found in both countries, but they were not always consistent. For example, while the perceived risk of dying was higher among rural residents in Ecuador (+3.1%), it was lower in Kenya (−2.3%) compared to urban residents, ceteris paribus. These findings suggest that individuals are aware of potential regional differences in the incidence of the disease and the importance of context (e.g., health infrastructure) in health outcomes.

## Limitations

This study has several limitations. First, the data were compiled at 1 point in time, and risk perceptions may have changed during the pandemic. Second, in both countries, the samples tended to be urban residents, more educated, and younger than the general population, which is likely attributable to the use of an online survey, as the only option available during the pandemic. Third, larger sample sizes would have allowed the perceived risks to be estimated with greater precision, statistical tests to have higher power, and offered the ability to conduct more analyses on population sub-groups. Fourth, the survey did not ask respondents about pre-existing conditions, which are also likely to affect risk perceptions. Fifth, the study is subject to non-response biases since participants did not respond to all the survey questions. Sixth, the results may be sensitive to the risk assessment measure used. Finally, the study did not consider the association between risk perceptions and health behaviors since they are likely to affect each other (i.e., risk perceptions affect health behaviors and the converse), complicating the regression analyses.

## Conclusions

The perceived risks of COVID-19-related infection, as well as perceptions of hospitalization and death in Kenya and Ecuador were significantly higher relative to reported statistics. No strong associations were found, in both countries, between perceived risk and age, which is a key factor in adverse health outcomes, including death, among COVID-19-infected individuals. Information about the risks of infection and adverse health outcomes is likely to influence individuals’ risk perceptions and ultimately their adoption of preventive measures. Thus, this information needs to be accurate and conveyed by trusted sources. Studies that assess the disease, risk perceptions, and associated factors provide information necessary to design population-wide health measures. Longer-term, these studies of COVID-19 risk perceptions in these countries could be used for retrospective research on the pandemic’s effects worldwide.

## Abbreviations

COVID-19: = Coronavirus Disease 2019
HRPP: = Human Research Protection Program
IHME: = Institute for Health Metrics and Evaluation
KES: = Kenyan Shillings
